# Efficacy of a Direct Aspiration First-Pass Technique (ADAPT) for Endovascular Treatment in Different Etiologies of Large Vessel Occlusion: Embolism vs. Intracranial Atherosclerotic Stenosis

**DOI:** 10.3389/fneur.2021.695085

**Published:** 2021-09-09

**Authors:** Geng Liao, Zhenyu Zhang, Guangzhi Zhang, Weijie Du, Chaomao Li, Hanxiang Liang

**Affiliations:** ^1^Department of Neurology, Maoming People's Hospital, Maoming, China; ^2^Department of Magnetic Resonance Imaging, Maoming People's Hospital, Maoming, China

**Keywords:** etiology, endovascular treatment, mechanical thrombectomy, embolism, intracranial atherosclerotic stenosis, stroke

## Abstract

**Background and Aims:** Aspiration thrombectomy is an effective method of recanalizing large vessel occlusion (LVO). However, the efficacy of a direct aspiration first-pass technique (ADAPT) for recanalization of LVO of different etiologies is not properly understood.

**Methods:** The prospectively collected database on ADAPT was reviewed retrospectively. We defined two groups of enrolled patients: the embolism-related occlusions (EMB-O) group and the intracranial atherosclerotic stenosis (ICAS)-related occlusion (ICAS-O) group. Baseline characteristics, procedural variables, and post-procedural variables were collected. Multivariate logistic regression analysis was used to identify first-pass recanalization predictors.

**Results:** Of 114 registered patients, 94 were eligible for this study (51 patients in the EMB-O group and 43 patients in the ICAS-O group). Achieving successful reperfusion immediately after direct aspiration was more frequent in the EMB-O group than in the ICAS-O group (64.71 vs. 27.91%, respectively, *p* = 0.006), with fewer additional rescue treatments needed (35.29 vs. 70.09%, respectively, *p* = 0.001). The EMB-O group also showed a higher final successful reperfusion rate (96.8 vs. 74.41%, *p* = 0.006). However, the 90-day good functional outcomes were not affected by the groups. Independent predictors of first-pass success of aspiration included the isolated middle cerebral artery site of occlusion, embolic etiology, and use of larger bore catheters.

**Conclusions:** The efficacy of ADAPT recanalization approach was better in EMB-O than in ICAS-O. In case of embolic etiology and the isolated MCA site of occlusion, using a larger aspiration catheter for direct aspiration thrombectomy may be reasonable.

## Introduction

A direct aspiration first-pass technique (ADAPT) as first-line therapy for stroke thrombectomy has shown non-inferior functional outcome at 90 days compared with stent retriever first-line thrombectomy (1). However, the efficacy of aspiration thrombectomy as a first-line approach for the recanalization of large vessel occlusions (LVOs) of different etiologies is not properly understood. There have been only a few studies about the endovascular treatment (EVT) of intracranial atherosclerotic stenosis (ICAS)-related LVO (ICAS-O) (2). They revealed that mechanical thrombectomy (MT) with a stent retriever or contact aspiration was less effective and more time-consuming in ICAS-O than in embolic LVO (EMB-O). In this study, we aimed to evaluate the efficacy of using ADAPT in LVO of different etiologies.

## Materials and Methods

### Study Populations

We performed a retrospective analysis of our prospectively gathered EVT database of patients with acute ischemic stroke (AIS). The intention was to identify patient cohorts in whom aspiration had been used as a first-line EVT approach in the anterior and posterior circulation (internal carotid artery, middle cerebral artery M1 and M2 segments, basilar artery, and vertebral artery in the V4 segment) and in whom the etiology of occlusion was identified. The database included all patients who presented with AIS due to LVO and who were treated with EVT between January 1, 2018, and June 30, 2020. The inclusion criteria were as follows:

- patients with intracranial large artery occlusions;
- underlying etiology classified as ICAS or embolism; and
- the onset time was defined as time from symptom onset to puncture ≤ 24 h. The onset time was defined as the last time when patient was still well.

Exclusion criteria encompassed the following:

- the etiology of LVO was classified as dissection, chronic total occlusion, moyamoya disease, vasculitis, or undetermined; and
- patients referred for pre-onset modified Rankin Scale (mRS) score > 2.

Patient demographics, comorbidities, premorbid functional status, conventional vascular risk factors, and laboratory findings assessed during admission, National Institutes of Health Stroke Scale (NIHSS), arterial occlusion site and lateralization, time from onset to puncture, Alberta Stroke Program Early CT Score (ASPECTS), intravenous thrombolysis before MT, angiographic, the number of passes with the aspiration device, time from puncture to reperfusion, and clinical data were collected. The location of initial occlusion site was determined using baseline computed tomography angiography or digital-subtraction angiography (DSA). Reperfusion performance was evaluated using the modified Thrombolysis in Cerebral Infarction grade (mTICI) grade (3). Successful reperfusion was defined as mTICI grade 2b or higher. First attempt recanalization (FAR) was defined as successful recanalization at the first attempt (4). All patients had a computed tomography (CT) at the end of the procedure and a CT or magnetic resonance image (MRI) scan 24 h after treatment onset to assess hemorrhagic complications. Intracerebral hemorrhages were classified in accordance with the European Cooperative Acute Stroke Study criteria (5). Subarachnoid hemorrhage (SAH) was classified using the modified Fisher scale (6). Symptomatic intracranial hemorrhage was defined as parenchymal hematoma type 2 using the ECASS III grading (European Cooperative Acute Stroke Study) according to imaging at 24 h, associated with an increase of at least four NIHSS points within 24 h, or resulting in death (7). New embolism in other vessels was defined as an occlusion of a previously unaffected non-downstream vascular territory observed on the angiogram after clot removal.

### Endovascular Procedure

In the current study, we divided patients into two groups based on the etiology of occlusion: the EMB-O group and ICAS-O group. ADAPT was used as the first-line approach for MT in both groups. In brief, a large-bore guide catheter such as a MPA1 0.088 inches (Cordis, USA) or 8-9F balloon guide catheter (Stryker, CA, USA) was advanced as far safely as possible into the internal carotid artery for anterior circulation thrombi and to the largest caliber vertebral artery in the posterior circulation thrombi. Then, the aspiration catheter was advanced and inserted into the internal thrombosis, usually over a microcatheter and micro guidewire. Dual aspiration was applied, and the clot was either ingested within the catheter or it was affixed at the catheter tip and then withdrawn under continuous vacuum exerted by a 50 or 60-ml syringe. If aspiration alone failed three times, access was maintained through the aspiration catheter, and then the second-line option was considered with the use of a stent retriever. Rescue treatments were allowed, including angioplasty, intra-arterial infusion of antithrombotics, and intracranial stenting. A control angiogram was performed to confirm reperfusion. Devices were selected at the discretion of neurointerventionalists based on the consensus of the stroke team.

### Etiologic Classification of Target Occlusive Lesions

The etiology of target LVO was determined by an independent core laboratory imaging analysis group based on medical history, angiography, and magnetic resonance imaging. Two experienced neurointerventionalists who were blinded to patient identification independently reviewed the clinical and image data of all the patients to determine the etiology of target LVO, and an experienced neurologist solved disparities. Interrater agreement for etiology of LVOs was assessed using Cohen's kappa coefficient (Cohen κ). Vasospasm caused by catheter and uncommon cerebral arterial diseases such as chronic total occlusion, dissection, and moyamoya disease were excluded. Etiological identification referred to TOAST criteria (Trial of Org 10172 in Acute Stroke Treatment) (8): if the occluded vessel was completely recanalized after primary thrombectomy, the etiology was classified as embolic occlusion, and a remnant stenosis >50% was classified as ICAS-O. In addition, the etiology of LVO in some patients was further evaluated by high-resolution magnetic resonance vascular wall analysis following MT during admission. Consequently, the EMB-O and ICAS-O groups were included in the analyses.

### Outcome Measures

The primary outcome was the rate of immediate and final successful reperfusion (mTICI score of 2b or 3) after the use of aspiration thrombectomy as the first-line approach; secondary outcomes included safety issues (procedural complications), procedural times (onset and femoral puncture to reperfusion), and 90-day all-cause mortality. Favorable outcome was defined by an modified Rankin Scale score (mRS) of 0 to 2 as evaluated by an independent senior vascular neurologist during face-to-face interviews or *via* telephone conversations at the 90th day of follow-up. Otherwise, since the clinical outcome may differ between the anterior and posterior circulation occlusions, the occlusion site as a subgroup was also included in the analysis of clinical outcomes.

### Statistical Analysis

Baseline characteristics and outcome measures were compared using univariate comparison and descriptive statistics. Variables were expressed as means ± standard deviations, medians (interquartile ranges), or numbers (percentages). Variables were compared using a non-parametric alternative for *t*-test (Mann-Whitney *U-*test) for non-continuous or non-normally distributed variables, Student's *t-*test for continuous variables, and χ^2^ test for categorical variables. Multivariate logistic regression was used to identify first-pass effect predictors in overall patients. *p*-values were two-tailed, and variables were considered significant at the < 0.05 level. Analysis was performed using PASWstat 18.0 (IBM Corporation, New York, USA).

## Results

**Figure 1:**
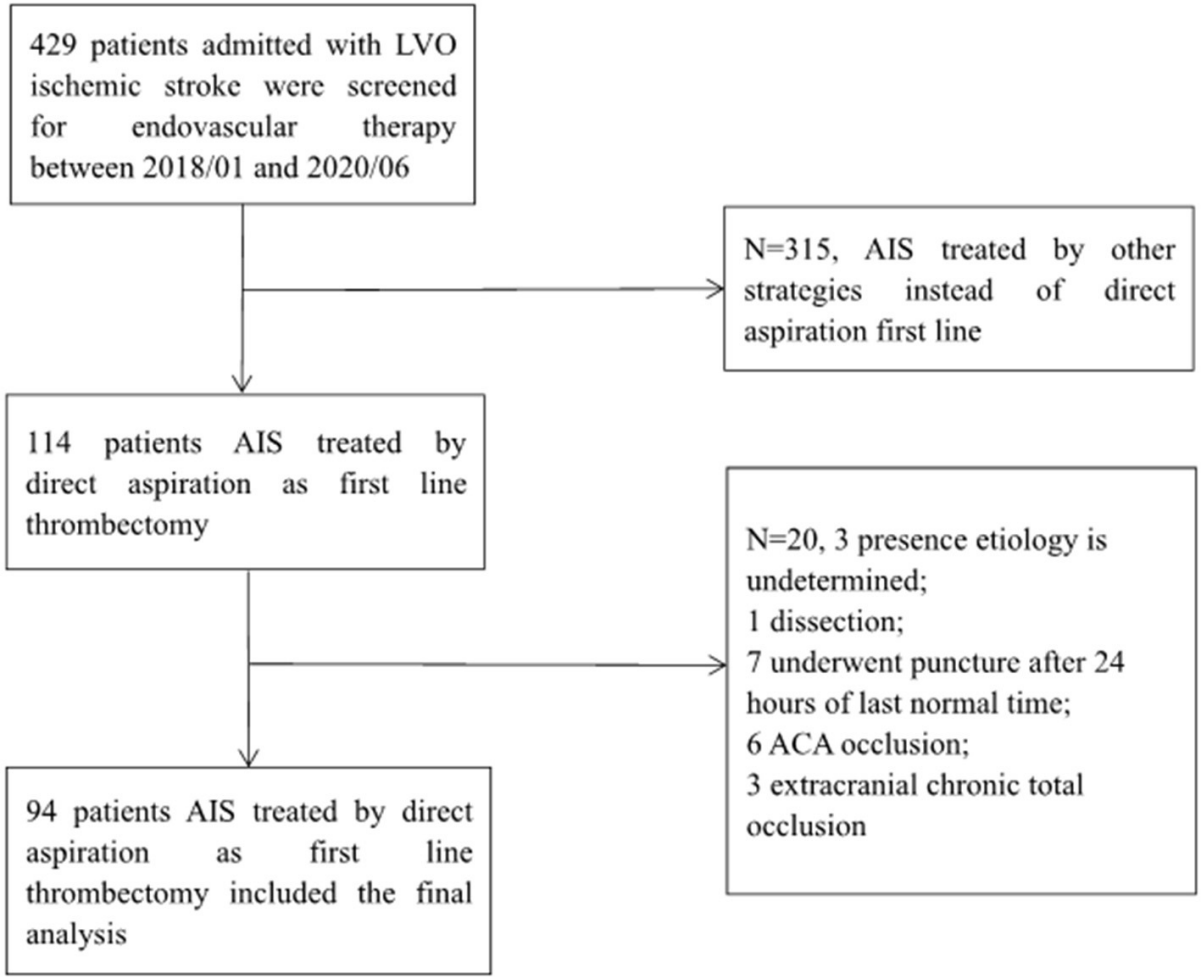
Patient inclusion flowchart. AIS, acute ischemic stroke; LVO, large vessel occlusion; ACA, anterior cerebral artery.

From January 2018 to June 2020, a total of 429 patients were screened for EVT of LVO ischemic stroke. Of these, 114 patients were treated with aspiration thrombectomy as first-line approach, and they constituted the study sample. Among them, 94 patients were included in the final analysis. The patient inclusion flowchart is shown in Figure 1. There was a good agreement on identification of the etiology of target LVO (Cohen κ: 0.871) between two independent neurointerventionalists. Overall, FAR and successful recanalization rates after simple contact aspiration were achieved in 44.68% (42/94) and 47.87% (45/94) of patients, respectively. The final successful recanalization rate was 86.17% (81/94).

### Baseline Characteristics

The characteristics of study sample are described in Table 1.

**Table 1:** Comparison of baseline characteristics in 94 patients with aspiration thrombectomy as first-line approach.

**Variable**	**Overall**	**Embolism-related occlusions**	**ICAS-related occlusions**	*p*-values^*^
No. of patients	94	51	43	
Age, years; mean ± SD	68.71 ± 13.33	68.82 ± 13.36	67.28 ± 11.20	0.307
Men	56 (59.6)	27 (52.9)	29 (67.4)	0.224
Hypertension	51 (54.3)	19 (37.3)	32 (74.4)	0.001
Diabetes mellitus	19 (20.2)	5 (9.8)	14 (32.6)	0.013
Dyslipidemia	12 (12.8)	3 (5.9)	9 (20.9)	0.062
Atrial fibrillation	39 (41.5)	37 (72.5)	2 (4.7)	0.000
Cardiovascular disease	18 (19.1)	10 (19.6)	8 (18.6)	1.000
Current smoking	8 (8.50)	4 (7.80)	4 (9.30)	1.000
**Site of occlusion**				
ICA siphon	28 (29.79)	18 (35.29)	10 (23.26)	NA
ICA siphon and MCA tandem	16 (17.02)	10 (19.61)	6 (13.95)	NA
Middle cerebral artery, M1	20 (21.28)	9 (17.65)	11 (25.58)	NA
Middle cerebral artery, M2	4 (4.26)	3 (5.88)	1 (2.33)	NA
Basilar artery	18 (19.15)	10 (19.61)	8 (18.60)	NA
Vertebral artery	8 (8.51)	0	8 (18.60)	NA
Initial NIHSS score, median (IQR)	14.95 (10–25)	15.73 (10–21)	14.30 (10–19)	0.345
ASPECTS, median (IQR)	8.44 (7–10)	8.0 (5–10)	8.91 (8–10)	0.078
Previous use of IV thrombolysis	15 (16.0)	10 (19.6)	5 (11.6)	0.441
Onset-to-door time, min, median (IQR)	256 (120–360)	224 (120–300)	293 (180–390)	0.040
Onset-to-puncture time, min, median (IQR)	353 (201–481)	320 (217–444)	394 (259–513)	0.029
The number of passes with the aspiration device (mean ± SD)	1.11 ± 0.373	1.10 ± 0.300	1.12 ± 0.448	0.815
Use of BGC	11	9	2	NA
Use of larger bore catheters (≥060)	29	23	6	NA
SOFIA PLUS 070	19	16	3	NA
Catalyst 060	8	6	2	NA
NAVIEN 072	2	1	1	NA
HR-MR following MT	19	2	17	NA

*Values expressed as n (%) unless otherwise indicated. NA, not applicable; ICAS, intracranial atherosclerotic stenosis; ASPECTS, Alberta Stroke Program Early CT Score; ICA, internal carotid artery; IQR, interquartile range; IV, intravenous; MCA, middle cerebral artery; NIHSS, National Institutes of Health Stroke Scale; HR-MR, high-resolution magnetic resonance vascular wall analysis; BGC, balloon guide catheter; MT, mechanical thrombectomy*.

* *p-values calculated using Student's t-test or Mann–Whitney U-test or χ^2^ test, as appropriate*.

Demographics and comorbidities showed that the risk factors for atherosclerosis, such as hypertension (74.4 vs. 37.3%; *p* = 0.001) and diabetes (32.6 vs. 9.8%; *p* = 0.013), were higher in the ICAS-O group than in the EMB-O group. The risk factors for atrial fibrillation in the EMB-O group were much more common than in the ICAS-O group (72.5 vs. 4.7%, respectively; *p* < 0.001). Time from onset to main hospital arrival and puncture time were shorter in the EMB-O group. Other demographic variables and comorbidities revealed no significant differences between the two groups.

### Effects of ADAPT for MT

Table 2 summarizes comparative results regarding the treatment.

**Table 2:** Procedure details.

**Variable**	**Embolism-related occlusions (*n* = 51)**	**ICAS-related occlusions (*n* = 43)**	***p*-values**
**Immediate effects following first-line thrombectomy of direct aspiration**			
mTICI 2b or greater on first pass of aspiration	29 (56.86)	13 (30.23)*	0.017
mTICI 2c-3 on first pass of aspiration	27 (51.94)	10 (23.25)	0.003
mTICI 2b or greater after aspiration	33 (64.71)	12 (27.91)	0.006
Time from groin puncture to first revascularization, min, median (IQR)	27 (20–30)	42 (30–60)	0.000
Rescue treatments after first-line thrombectomy of direct aspiration	18 (35.29)	31 (70.09)	0.001
Switching to stent retriever device	18 (35.29)	11 (25.58)	0.429
Balloon angioplasty	1 (1.96)	18 (41.86)	0.000
Permanent stenting	2 (3.92)	10 (23.26)	0.013
Tirofiban/Eptifibatide infusion	18 (35.29)	25 (58.14)	0.344
**Final endovascular treatment results**			
Final mTICI flow [*n* (%)]			
2b−3	49 (96.8)	32 (74.41)	0.006
0–2a	2 (3.92)	11 (25.58)	0.000
Time from groin puncture to final revascularization, min, median (IQR)	42 (29–50)	83 (60–103)	0.000
**Complications**			
Hemorrhage, all types	6 (11.76)	3 (6.98)	0.876
Hemorrhage, PH2	3 (5.88)	2 (4.65)	0.791
SAH, grade 3 or 4	1 (1.96)	1 (2.33)	0.903
New embolism in other vessels	6 (11.76)	2 (4.65)	0.546
Iatrogenic dissection or rupture	0	1 (2.33)	0.932
Spasm	3 (5.88)	2 (4.65)	0.791

Data presented as n (%, 95% confidence interval), median (95% confidence interval), n (%), or mean (SD). mTICI, modified thrombolysis in cerebral infarction; NIHSS, National Institutes of Health stroke scale; IQR, interquartile range; PH2, parenchymal hematoma type 2; SAH, subarachnoid hemorrhage. *One patient was treated successfully by aspiration, but due to re-occlusion after 10 min, another round of rescue therapy was required.

Successful reperfusion after first pass of aspiration was more common in the EMB-O group than in the ICAS-O group (56.86 vs. 30.23%, *p* = 0.017). Immediate successful reperfusion after aspiration was achieved more frequently in the EMB-O group (64.71%) than in the ICAS-O group (27.91%; *p* = 0.006), as well as the complete reperfusion (mTICI 2C-3) rate (51.94 vs. 23.26%, *p* = 0.003). The rate of total switching to another thrombectomy device was more frequent in the ICAS-O group than in the EMB-O group (70.09 vs. 35.29%, *p* = 0.001). As rescue treatments, balloon angioplasty and permanent stenting were performed more frequently in the ICAS-O group. Tirofiban/eptifibatide infusion was performed in both groups with no significant difference.

The final successful reperfusion rate was higher in the EMB-O group than in the ICAS-O group (EMB-O, 96.8% vs. ICAS-O, 74.41%; *p* = 0.006). The time from puncture to final revascularization was longer in the ICAS-O group.

Intracerebral hemorrhagic transformation of any type and parenchymal hematoma type 2 occurred at similar rates in the two groups. The occurrence of new embolism in other vessels and iatrogenic dissection or rupture were similar between the two groups.

### Clinical Outcomes After EVT

**Figure 2:**
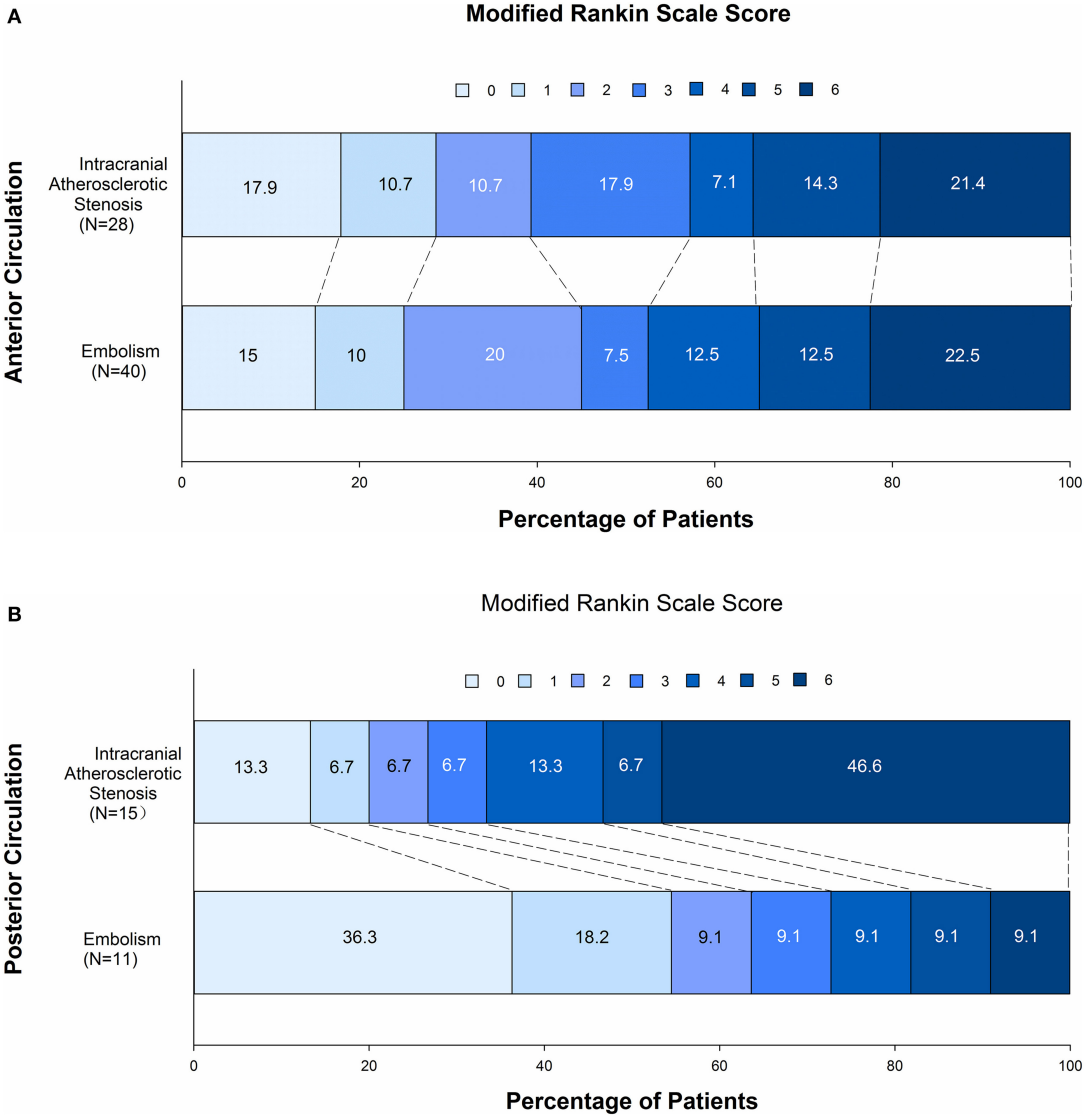
Comparison of clinical outcome at 90 days after a direct aspiration first-pass technique (ADAPT) for the endovascular treatment of stroke in embolism-related occlusions and intracranial atherosclerotic stenosis-related occlusions in the anterior circulation **(A)** and in the posterior circulation **(B)**.

There was no significant difference in favorable clinical outcome at 3 months (Table 3). To investigate the clinical outcome, we divided patients into two subgroups based on the location of the occlusion (anterior circulation vs. posterior circulation). The subgroup analysis showed that the location of the occlusion did not affect good clinical outcome at 3 months (Table 3, Figure 2A). However, 90-day mortality in patients with occlusion in the posterior circulation was higher in the ICAS-O than in the EMB-O group (16.28 vs. 1.96%, *p* = 0.012; Table 3, Figure 2B).

**Table 3:** Clinical outcomes at 90 days after endovascular treatment.

	**Embolism-related occlusions (*n* = 51)**	**ICAS-related occlusions (*n* = 43)**	**OR (95% CI)**	***p*-values**
mRS 0–2 at 90 days	24 (47.06)	15 (34.88)	1.659 (0.721–3.821)	0.233
Anterior circulation	18 (35.29)	11 (25.58)	1.587 (0.649–3.879)	0.310
Posterior circulation	6 (11.76)	4 (9.30)	1.064 (0.281–4.022)	0.928
Mortality at 90 days	10 (19.61)	13 (30.23)	0.563 (0.218–1.455)	0.233
Anterior circulation	9 (17.65)	6 (13.95)	1.321 (0.43–4.064)	0.626
Posterior circulation	1 (1.96)	7 (16.28)	0.101 (0.012–0.856)	0.012

*Data presented as n (%) or median (IQR). OR, odds ratio; mRS, modified Rankin Scale; CI, confidence interval; ICAS, intracranial atherosclerotic stenosis*.

### Predictors of FAR Using ADAPT

To investigate predictors of FAR, multivariate logistic regression analysis was applied in all cases of the two groups. Embolic etiology, isolated MCA occlusion, and the use of larger aspiration catheters were predictors of FAR (Table 4). Multivariate logistic regression analysis was applied in all cases of the two groups.

**Table 4:** Multivariate regression analysis for predictors of first attempt recanalization of ADAPT.

Variable^*^	**OR**	**95% CI**	***p*-values**
Age	0.997	0.956–1.039	0.871
Male gender	0.585	0.196–1.743	0.336
Diabetes	1.870	0.487–7.181	0.362
Hypertension	1.201	0.330–4.263	0.777
Hyperlipidemia	0.970	0.207–4.556	0.969
Pre-stroke mRS score	0.753	0.038–14.802	0.753
Baseline NIHSS score	0.939	0.865–1.019	0.133
IV tPA	1.764	0.397–7.846	0.456
Onset-to-puncture time	1.001	0.998–1.004	0.364
Site of occlusion			
Isolated MCA	4.506	1.066–19.045	0.041
Isolated VBA	3.5	0.887–13.807	0.074
Isolated ICA	0.560	0.194–0.615	0.284
Tandem occlusions	0.313	0.057–1.721	0.182
Etiology: embolism	3.505	1.031–11.917	0.045
Use of larger bore catheters (≥060)	9.167	2.6–32.322	0.001

* *Variables showing statistical significance (p < 0.05) are shown in bold font. ADAPT, a direct aspiration first-pass technique; IV tPA, intravenous tissue plasminogen activator; mRS, modified Rankin Scale; NIHSS, National Institutes of Health Stroke Scale; MCA, middle cerebral artery; VBA, vertebral basilar artery; ICA, internal carotid artery*.

## Discussion

Our study demonstrates that ADAPT is more successful to achieve full recanalization in embolic LVO compared to intracranial atherosclerotic stenosis LVO. Additionally, we also found that independent predictors of FAR of aspiration included the isolated middle cerebral artery site of occlusion, embolic etiology, and use of larger bore catheters. Subgroup analysis showed that trends for the mortality of ICAS-O patients were higher than those of EMB-O in the posterior circulation.

ICAS is one of the main causes of acute stroke in Asian, Hispanic, and African populations (9). Furthermore, some studies have documented that ICAS-O is responsible for ~12–34% of all causes of LVO in east Asia (2, 10) and 1.9%−5.5% in Western countries (11, 12). It has been shown that the pathomechanism of ICAS-O is likely due to *in situ* thromboocclusion (13). Some studies have shown that stent retriever thrombectomy for obtaining initial recanalization is equally effective in ICAS-O and EMB-O, although reocclusion is frequent after an initial recanalization in ICAS-O (2). Unlike stent retriever thrombectomy, aspiration first seemed less effective for the recanalization of ICAS-O (14).

In this cohort, baseline characteristics of the two groups were different in many risk factors for stroke, such as hypertension, diabetes mellitus, and atrial fibrillation, which stems from the fact that the grouping was based on etiology. It is noteworthy that the onset-to-door time in the ICAS-O group was much higher than in the EMB-O group. Indeed, neurologic deficit at onset was mild in some ICAS-O cases, which, along with insufficient health education, resulted in delayed visit to hospital. Underlying ICAS was identified in approximately a third of patients, which is consistent with the previous reports in Asia (2, 10). The primary outcomes of this study, immediate (56.86%) and final reperfusion (96.8%) performance, were better in the EMB-O group. In addition, time from groin puncture to final revascularization in the EMB-O group (42 min) was significantly lower than in the ICAS-O group (83 min). Interestingly, the higher rescue treatment (70.09%) involving the use of other devices in the ICAS-O group matches those from the other reports (40–59.7%) (2, 14, 15). It is suggested that ICAS-O may require a different first-line treatment strategy compared with a direct aspiration thrombectomy.

MT may cause vessel damage, which has been confirmed by clinical and animal studies (16, 17). In the current study, there were no inter-group differences in all and severe complications. However, new embolism to previously uninvolved territory (8.5%) was more frequent in this study than in previous reports (1, 14, 15). This may be attributed to the lesser application of the balloon guide catheter (11.7%), and associated with histological clot composition.

Despite many disadvantages in the ICAS-O compared with the EMB-O group, 3-month favorable outcomes and mortality did not differ between the groups. The enhanced lateral circulation of atherosclerotic stenosis occlusion and ischemic preconditioning compared to embolic occlusion may play crucial roles, as the ASPECTS in the ICAS-O group was higher than that of EMB-O (8.9 vs. 8.0), indicating the better collaterals in the ICAS group, though it may not be statistically significant. Of note, this study included the patients with posterior circulation LVO. It is not clear whether revascularization of posterior circulation occlusion can benefit from EVT (18, 19). Therefore, the clinical outcome was further analyzed in two subgroups separately (anterior and posterior circulation groups). To our surprise, although there was no difference in good outcomes between the two subgroups, we revealed that mortality was significantly higher in the posterior circulation subgroup of ICAS-O than EMB-O. Although the exact mechanism has not been illuminated, it is likely that shorter recanalization time plays a role here.

Successful recanalization using ADAPT depends on multiple factors such as location of the occlusion, vascular anatomy, clot characteristics, device characteristic, and underlying etiology (4, 20). Previous studies have shown that clots from cardioembolism had a significantly higher proportion of erythrocyte and a lower proportion of fibrin compared with those from large-artery atherosclerosis, and erythrocyte components were positively related to successful reperfusion (21–23). In our study, the presence of embolic etiology, isolated MCA occlusion, and the use of larger aspiration catheters were independent predictors of FAR with aspiration first-line approach. In contrast, underlying atherosclerosis has been shown to be a predictor of unsuccessful recanalization. Given that ACE catheters have not yet been allowed for application in our stroke center, we mainly use the Sofia catheter as a primary aspiration catheter. Sofia catheter is a distal aspiration catheter with a specific hybrid design. Its braid and coil construction combines different softness segments with a distal inner lumen of 0.055–0.070 inches in 5F and 6F Plus versions, respectively (24). The safety and efficacy of the Sofia aspiration catheter in a large population with first-line use has been reported (25). Our experience shows that a large inner lumen aspiration catheter such as Sofia 6F PLUS (0.070 inches) works better than a small one. Besides we found that the embolic etiology of LVO was a positive FAR predictor with direct aspiration thrombectomy, which may be a useful point for clinical reference.

The limitations of this study were as follows. First, since this was a single-center study, the sample size and representativeness are insufficient. Second, it is difficult to determine whether ICAS was really the cause of LVO. Although we had collected the clots in this study, we are not yet providing histopathological analysis due to technical limitations, which requires further research. Thus, the diagnosis of underlying ICAS after thrombectomy mainly depended on the imaging characteristics of the local lesion during the procedure. Third, we used an unblinded approach for the evaluation of NIHSS and mRS. At present, due to time constraints, it is difficult to design a randomized controlled study based on the etiology of LVO stroke before intervention. Future prospective and multicentric studies with blinded endpoint registration are warranted.

## Conclusions

The evaluation of the efficacy of ADAPT showed higher immediate and final reperfusion in EMB-O than in ICAS-O. However, there were no significant differences in the 90-day outcomes between the two etiological groups of LVO. In case of embolic etiology and isolated MCA site of occlusion, using a larger aspiration catheter pretreatment for direct aspiration thrombectomy may be reasonable.

## Data Availability Statement

The original contributions presented in the study are included in the article/Supplementary Material, further inquiries can be directed to the corresponding author/s.

## Ethics Statement

The studies involving human participants were reviewed and approved by The Institutional Ethics Committee of Maoming People's Hospital. They granted approval for our work (2019027). The patients/participants provided their written informed consent to participate in this study.

## Author Contributions

GL designed the report, completed the statistical analysis, wrote the protocol, wrote the first draft of the manuscript, and took overall responsibility. GL, ZZ, WD, CL, and GZ contributed to the acquisition of data. GL, ZZ, and HL managed the literature searches and analyses. All authors have provided a substantial contribution to the study.

## Funding

This work was supported by grants from the High-level Hospital Construction Research Project of Maoming People's Hospital (Reference No. Yueweihan 2018413) and the Special Fund of Science and Technology of Guangdong Province, China (Reference No. 2020S00050).

## Conflict of Interest

The authors declare that the research was conducted in the absence of any commercial or financial relationships that could be construed as a potential conflict of interest.

## Publisher's Note

All claims expressed in this article are solely those of the authors and do not necessarily represent those of their affiliated organizations, or those of the publisher, the editors and the reviewers. Any product that may be evaluated in this article, or claim that may be made by its manufacturer, is not guaranteed or endorsed by the publisher.

## References

[1] Turk AS Siddiqui A Fifi JT De Leacy RA Fiorella DJ Gu E. Aspiration thrombectomy versus stent retriever thrombectomy as first-line approach for large vessel occlusion (COMPASS): a multicentre, randomised, open label, blinded outcome, non-inferiority trial. Lancet. (2019) 393:998–1008. 10.1016/S0140-6736(19)30297-1 30860055

[2] Park H Baek JH Kim BM. Endovascular treatment of acute stroke due to intracranial atherosclerotic stenosis–related large vessel occlusion. Front Neurol. (2019) 10:308. 10.3389/fneur.2019.00308 31001193 PMC6454085

[3] Tomsick T Broderick J Carrozella J Khatri P Hill M Palesch Y. Revascularization results in the interventional Management of Stroke II trial. AJNR Am J Neuroradiol. (2008) 29:582–7. 10.3174/ajnr.A0843 18337393 PMC3056457

[4] Anadani M Alawieh A Vargas J Chatterjee AR Turk A Spiotta A. First attempt recanalization with ADAPT: rate, predictors, and outcome. J Neurointerv Surg. (2018) 2018:1–6. 10.1136/neurintsurg-2018-014294 30530772

[5] Fiorelli M Bastianello S von Kummer R del Zoppo GJ Larrue V Lesaffre E. Hemorrhagic transformation within 36 hours of a cerebral infarct: relationships with early clinical deterioration and 3-month outcome in the European cooperative acute stroke study I (ECASS I) cohort. Stroke. (1999) 30:2280–4. 10548658 10.1161/01.str.30.11.2280

[6] Frontera JA Claassen J Schmidt JM Wartenberg KE Temes R Connolly ES. Prediction of symptomatic vasospasm after subarachnoid hemorrhage: the modified fisher scale. Neurosurgery. (2006) 59:21–7. 10.1227/01.Neu.0000218821.34014.1b 16823296

[7] Wahlgren N Ahmed N Dávalos A Ford GA Grond M Hacke W. SITS-MOST Investigators. Thrombolysis with alteplase for acute ischaemic stroke in the Safe Implementation of Thrombolysis in Stroke-Monitoring Study (SITS-MOST): an observational study. Lancet. (2007) 369:275–82. 10.1016/S0140-6736(07)60149-4 17258667

[8] Adams HP Jr Bendixen BH Kappelle LJ Biller J Love BB Gordon DL. Classification of subtype of acute ischemic stroke. Definitions for use in a multicenter clinical trial. TOAST. Trial of Org 10172 in acute stroke treatment. Stroke. (1993) 24:34–41. 10.1161/01.str.24.1.35 7678184

[9] Wong LK. Global burden of intracranial atherosclerosis. Int J Stroke. (2006) 1:158–9. 10.1111/j.1747-4949.2006.00045.x 18706036

[10] Jia B Feng L Liebeskind DS Huo X Gao F Ma N. Mechanical thrombectomy and rescue therapy for intracranial large artery occlusion with underlying atherosclerosis. J NeuroIntervent Surg. (2017) 2017:1–6. 10.1136/neurintsurg-2017-013489 29203731

[11] Gascou G Lobotesis K Machi P Maldonado I Vendrell JF Riquelme C. Stent retrievers in acute ischemic stroke: complications and failures during the perioperative period. AJNR Am J Neuroradiol. (2014) 35:734–40. 10.3174/ajnr.A3746 24157734 PMC7965801

[12] Dobrocky T Kaesmacher J Bellwald S Piechowiak E Mosimann PJ Zibold F. Stent-retriever thrombectomy and rescue treatment of M1 occlusions due to underlying intracranial atherosclerotic stenosis: cohort analysis and review of the literature. Cardiovasc Intervent Radiol. (2019) 42:863–72. 10.1007/s00270-019-02187-9 30859286

[13] Baek JH Kim BM Kim DJ Heo JH Nam HS Song D. Importance of truncal-type occlusion in stentriever-based thrombectomy for acute stroke. Neurology. (2016) 87:1542–50. 10.1212/WNL.0000000000003202 27629085

[14] Yoo J Lee SJ Hong JH Kim YW Hong JM Kim CH. Immediate effects of first-line thrombectomy devices for intracranial atherosclerosis-related occlusion: stent retriever versus contact aspiration. BMC Neurol. (2020) 20:283–92. 32682406 10.1186/s12883-020-01862-6 PMC7368707

[15] Lapergue B Blanc R Guedin P Decroix JP Labreuche J Preda C. A direct aspiration, first pass technique (ADAPT) versus stent retrievers for acute stroke therapy: an observational comparative study. AJNR Am J Neuroradiol. (2016) 37:1860–5. 10.3174/ajnr.A4840 27256852 PMC7960455

[16] Liao G Zhang Z Che X Liang H. Mechanical thrombectomy using a stent retriever with an intermediate catheter for partially occluded middle cerebral artery fenestration. World Neurosurg. (2020) 138:355–9. 10.1016/j.wneu.2020.03.096 32224267

[17] Koge J Kato S Hashimoto T Nakamura Y Kawajiri M Yamada T. Vessel wall injury after stent retriever thrombectomy for internal carotid artery occlusion with duplicated middle cerebral artery. World Neurosurg. (2019) 123:54–8. 10.1016/j.wneu.2018.11.223 30529524

[18] Liu X Dai Q Ye R Zi W Liu Y Wang H. Endovascular treatment versus standard medical treatment for vertebrobasilar artery occlusion (BEST): an open-label, randomised controlled trial. Lancet Neurol. (2020) 19:115–22. 10.1016/S1474-4422(19)30395-3 31831388

[19] Siebert E Bohner G Zweynert S Maus V Mpotsaris A Liebig T. Revascularization techniques for acute basilar artery occlusion: technical considerations and outcome in the setting of severe posterior circulation steno-occlusive disease. Clin Neuroradiol. (2019) 29:435–43. 10.1007/s00062-018-0683-3 29651586

[20] Blanc R Redjem H Ciccio G Smajda S Desilles JP Orng E. Predictors of the aspiration component success of a direct aspiration first pass technique (ADAPT) for the endovascular treatment of stroke reperfusion strategy in anterior circulation acute stroke. Stroke. (2017) 48:16149. 10.1161/STROKEAHA.116.016149 28428348

[21] Kamalian S Morais LT Pomerantz SR Aceves M Sit SP Bose A. Clot length distribution and predictors in anterior circulation stroke: implications for intra-arterial therapy. Stroke. (2013) 44:3553–6. 10.1161/STROKEAHA.113.003079 24105699 PMC3927722

[22] Kim SK Yoon W Kim TS Kim HS Heo TW Park MS. Histologic analysis of retrieved clots in acute ischemic stroke: correlation with stroke etiology and gradient-echo MRI. AJNR Am J Neuroradiol. (2015) 36:1756–62. 10.3174/ajnr.A4402 26159515 PMC7968760

[23] Hashimoto T Hayakawa M Funatsu N Yamagami H Satow T Takahashi JC. Histopathologic analysis of retrieved thrombi associated with successful reperfusion after acute stroke thrombectomy. Stroke. (2016) 47:3035–7. 10.1161/STROKEAHA.116.015228 27780903

[24] Wong JHY Do HM Telischak NA Moraff AM Dodd RL Marks MP. Initial experience with SOFIA as an intermediate catheter in mechanical thrombectomy for acute ischemic stroke. J Neurointerv Surg. (2017) 9:1103–6. 10.1136/neurintsurg-2016-012750 27789787

[25] Marnat G Barreau X Detraz L Bourcier R Gory B Sgreccia A. First-line sofia aspiration thrombectomy approach within the endovascular treatment of ischemic stroke multicentric registry: efficacy, safety, and predictive factors of success. Am J Neuroradiol. (2019) 40:1006–12. 10.3174/ajnr.A6074 31122921 PMC7028582

